# Jefferson Medical College

**Published:** 1844-06-01

**Authors:** Edward R. Squibb


### CLINICAL LECTURES AND REPORTS.

### JEFFERSON MEDICAL COLLEGE.

*April* 10, 1844.

### CLINIC OF PROFESSOR MÜTTER.

[Reported by Edward R. Squibb.]

Inasmuch as the blind opposition of some surgeons has so much condemned the practice of Plastic surgery,—stigmatizing it as a cruel and useless multiplication of operations which are never heard of after,—it is desirable that the class should test its utility by results, and judge for themselves; there never having been a single case in which he had operated, designedly withdrawn from notice, the Professor wished therefore to call the attention of the class for a moment to

Case I.—That of A. D., æt. 19, who in a fight had lost the right ala of his nose, for the relief of which deformity he presented himself here. This was effected by an entirely new operation, as described at the time, (see report of last lecture,) and he is now presented for the purpose of exhibiting the result, which, even in its yet unfinished condition, presents but a very slight trace of the deformity; union by first intention having taken place throughout, without the intervention of any bad symptom whatever.

Case II.—H. æt. 14. Presents himself for the purpose of ascertaining whether anything can be done for the relief of his deformity.

This has evidently been a case of coxalgia; cured in the third stage, by the establishment of anchylosis with the limb in a semi-flexed position. Can any thing be done for such a case? First examine the limb carefully by placing one hand upon the pelvis, and with the other grasping the limb, attempt to make slight flexion and extension; if any motion, however slight, should be produced, we have only false anchylosis, and the limb, by the aid of proper apparatus, (Humbolt's,) may be straightened, and made so far useful. But if on the other hand, as in this case, the pelvis is found to move with the limb, let it alone; you can do it no good. If you put on the apparatus here, and by means of your screw rupture the bony anchylosis which has formed, you inevitably bring back the disease and implicate the life of your patient. We shall therefore advise this lad to get a high-heeled shoe and a cane, and do the best he can with his leg in the bent position.

Case III.—Patrick P., æt. 9. Has a scrofulous tumour involving several glands in the neck. The diagnosis in these cases is by this time so familiar, that it will be unnecessary to take up time in going over it; I will therefore only add, that these tumours sometimes point of themselves, thereby precluding the necessity of an operation; at others, however, they are found to burrow under the fascia of the neck, forming large collections of pus. To obviate this difficulty, it is always best to open them as early as practicable after the deposite has taken place; in doing this the incision should always be made transversely, in the line of one of the natural folds of the neck; this avoids all deformity which would otherwise occur from the conspicuous position of the resulting cicatrix.

The older surgeons, indeed as late as the late Mr. Abernethy, had a great dread of opening such abscesses, owing to the supposed liability to inflammation from the introduction of air into the cavity; to prevent which, they used needles, making small valvular openings; but this method has now given place to that of opening them by incision. As we know that no serious consequences are likely to result, we will order this to be dressed with a simple poultice, and place the patient under the treatment which has become almost routine in such cases, namely, iod. of potassium 3 grains, three times a day in a little comp. syr. of sarsaparilla.

Case IV.—Jacob H., æt. 14. Has an abscess discharging from an orifice in the lumbar region of the back. This, although apparently so near the spinal column, does not involve it in the least degree. It is evidently an abscess, whose extent, as indicated by the great depth to which the probe passes, is many inches outwards and forwards, dissecting up the peritoneum from the muscles, being influenced in its direction to the surface of the back, by the peritoneum which exercises in these cases the same office for the viscera which it surrounds that the periosteum does for the bone, both being often instrumental in the preservation of their contents from diseases involving the surrounding tissues.

This case has been under treatment by poultices of elm bark externally, and iod. of potassium as an alterative internally. The former will be continued, but the latter changed for the solution of iod. of iron, five drops three times a day.

Case V.—J. C., æt. 5. A case of double scrotal and umbilical hernia.

Having had occasion so often to speak on hernia before the present class, I shall not now enter upon it minutely, wishing to make familiar to all the mode of reduction, and the application of the trusses, remarking, *en passant*, that such scrotal hernias are liable to be mistaken for varicocele, enlarged testes, &c., therefore requiring some caution; also in the application of the instruments to get the pad in the proper position, to avoid making pressure upon the spermatic cord, and also the supervention of concealed hernia, the liability to either of which is precluded by placing the pad carefully over the internal abdominal ring.

Case VI.—Leah S., æt. 8. Hare-lip, with fissure in the hard palate, a case presenting unusual deformity in appearance, as well as great inconvenience in speech, &c. In cases where the fissure extends through the hard palate, it is always proper to close the lips only, at an early age; the pressure made by tightening the integument over the maxilla being often sufficient to effect the closure of the cleft without the necessity of a further operation; leaving the more difficult one of staphyloraphy or stapheloplasty, until the tissues shall have had time to become more firm, and therefore better adapted to the success of the operation; but more particularly should it be deferred, that the patient on arriving at years of discretion, say 16 or 17, may exercise a choice, after being able to appreciate the pain and value of the operation. The operation for simple hare-lip should be performed at as early an age as practicable, provided the child be strong and healthy; in cases of weak and sickly children, however, it must be deferred, as the blood does not in such cases contain a sufficiency of fibrin to ensure its success. I have seen many such cases in which it had been performed, where, on removing the pins, the edges separated like wet paper.

In the one now before us, the first step is to break up the adhesions which have formed between the lip and gum, by which we are enabled to bring the lip and septum narium into their proper places;—the second, to freshen the edges of the fissure, making them square and even, up to the point at which we wish to limit the union, not being afraid of taking off too much, as there is plenty of tissue, (the curvilinear modification of Dr. Rhea Barton not being at all necessary in this case.) This accomplished, we next proceed to place the pins; I prefer those made by tipping common cambric needles with sealing wax, to the ordinary hare-lip pins of the shops, as being easier of insertion, and easier to manage afterwards; these are surrounded by figure 8 turns of the ligature, passing a turn or two of each ligature over the neighbouring pin to compress the intermediate parts, knotting each firmly.

Case VII.—Edward M., æt. 27. Has a large tumour under the sterno-cleido-mastoid muscle and fascia of the neck. In every case like the present, it is the duty of the surgeon to make every effort to discuss,—as the older surgeons would say,—the tumour, before subjecting the patient to the inconvenience and hazard of an operation of such extent as would be required in the case before us. Such means have been used here, but with the sole effect of softening the tumour a little, without in any degree checking the rapidity of its growth, which is such as now to require more effectual means for the relief of the patient. Before resorting to a cruel operation for the removal of a tumour of doubtful character, it is always proper to use a grooved needle for the purpose of ascertaining what may be its contents; thereby you may often save the patient from much unnecessary pain, and yourselves from learning this lesson in a less agreeable manner at some future day.

This small instrument, which gives the patient little pain, shows to us that this is but a sac of pus, bound down by the muscles and fascia of the neck; and that instead of a very severe and extensive operation, we shall only require a small valvular opening to evacuate the pus, which as you see flows quite freely, and the tumour immediately disappears; we shall have it dressed with an ordinary poultice, and order the patient to continue the use of the liq. ferri iod., three times a day.

Case VIII.—Mrs. ——'s child. With this case of chronic hydrocephalus, I will not detain you, as the time has already expired,—but merely remark that it will be placed under the new treatment which has been proposed for the relief of this affection, and exhibited at some future clinic.

